# A Novel Antimicrobial Peptide Displaying Broad-Spectrum Activity Against Pan-Resistant Pathogens and Low Propensity for Resistance Development

**DOI:** 10.3390/antibiotics15080813

**Published:** 2026-08-20

**Authors:** Betul Zehra Temur, Esma Bolat, Meltem Ayas, Sengül Nisa Demirci, Nihan Unubol, Neval Yurttutan Uyar, Ozge Can, Tanil Kocagoz

**Affiliations:** 1 Department of Biomedical Engineering, Graduate School of Natural and Applied Sciences, Acibadem University, Istanbul 34752, Türkiye; betulzkarakus@gmail.com; 2 Department of Medical Biotechnology, Graduate School of Health Sciences, Acibadem University, Istanbul 34752, Türkiye; esmaaybakan@hotmail.com; 3 Medical Laboratory Techniques, Vocational School of Health Services, Acibadem University, Istanbul 34752, Türkiye; meltem.ayas@acibadem.edu.tr (M.A.); nihan.unubol@acibadem.edu.tr (N.U.); 4 Department of Medical Microbiology, School of Medicine, Acibadem University, Istanbul 34752, Türkiye; neval.uyar@acibademlabmed.com.tr; 5 Department of Biomedical Engineering, Faculty of Engineering and Natural Sciences, Acibadem University, Istanbul 34752, Türkiye; sengul.demirci@live.acibadem.edu.tr; 6 Acibadem Labmed Clinical Laboratory, Istanbul 34752, Türkiye

**Keywords:** peptide antibiotics, antimicrobial peptide, protease resistance, multi-drug resistant bacteria, ESKAPE pathogens, *E. coli*

## Abstract

Objectives: The escalation of multidrug-resistant (MDR) clinically relevant bacterial isolates, including *Escherichia coli* and key ESKAPE pathogens, represents a critical global healthcare threat. While antimicrobial peptides (AMPs) offer promising alternatives, metabolic instability often limits their clinical use. This study investigated the therapeutic potential, antimicrobial efficacy, and resistance dynamics of peptide D-TN6 against highly resistant bacterial strains. Methods: D-TN6 efficacy was evaluated against 164 clinical isolates, including pan-drug-resistant, carbapenem-resistant, and polymyxin-resistant phenotypes. A 20-passage serial induction assay compared resistance development kinetics of D-TN6 against gentamicin over an extended period. Results: D-TN6 demonstrated potent efficacy against MRSA (minimum inhibitory concentration (MIC)_90_: 1 µg/mL) and remained effective against polymyxin B-resistant *Klebsiella pneumoniae* (MIC_90_: 8 µg/mL) and pan-drug-resistant *Acinetobacter baumannii* (MIC_90_: 8 µg/mL). In resistance assays, while the gentamicin MIC increased 256-fold by the 17th passage, the D-TN6 MIC remained constant at 1 µg/mL throughout the 20-passage study. Conclusions: These findings underscore D-TN6 as a robust therapeutic candidate. Its efficacy against MDR strains and its feature of low propensity for resistance development—likely due to membrane disruption—position D-TN6 as a promising solution for life-threatening infections where conventional last-resort agents fail.

## 1. Introduction

MDR microorganisms pose a significant threat to global health, and their increasing prevalence complicates the treatment of infectious diseases with conventional antimicrobial drugs [1,2]. According to the World Health Organization (WHO) and recent global burden analyses, antimicrobial resistance (AMR) represents a major public health crisis. The landmark 2019 Global Burden of Disease (GBD) study reported an estimated 1.27 million deaths directly attributable to bacterial AMR and 4.95 million associated deaths [3]. Predictions indicate that if new therapeutics or treatments are not developed, AMR-associated mortality could reach 10 million deaths annually, with an estimated economic burden of $100 trillion by 2050 [2]. The emergence of AMR is further exacerbated by inadequate infection control in hospitals, and socio-economic factors such as the accumulation of antibiotics in the environment and their transmission through the food industry [4].

As a leading Gram-negative pathogen, *Escherichia coli* represents one of the primary drivers of global antimicrobial resistance-attributable mortality, accounting for over 800,000 direct and associated deaths worldwide in 2019 [3]. To highlight the urgent global threat, the World Health Organization (WHO) established a priority list of bacterial pathogens, categorizing them into critical-, high-, and medium-priority groups [4,5]. The critical group includes carbapenem-resistant *A. baumannii* and *Enterobacteriaceae* (featuring *E. coli* as a major representative). Carbapenem-resistant *P. aeruginosa*, vancomycin-resistant *E. faecium* (*VRE*), and methicillin-resistant *S. aureus* (*MRSA*) belong to the high-risk group in the 2024 WHO Bacterial Priority Pathogens List. Other multidrug-resistant pathogens are collectively referred to by the acronym ESKAPE (*E. faecium*, *S. aureus*, *K. pneumoniae*, *A. baumannii*, *P. aeruginosa*, and *Enterobacter* species), which represent the highest priority threats to public health [6]. ESKAPE pathogens are associated with diverse clinical infections and alarming mortality rates, ranging from 30% in *VRE* bacteremia to as high as 70% in carbapenem-resistant *K. pneumoniae* bloodstream infections. These pathogens exhibit resistance to oxazolidinones, lipopeptides, macrolides, fluoroquinolones, tetracyclines, β-lactams, β-lactam–β-lactamase inhibitor combinations, and last-line antibiotics, including carbapenems, glycopeptides, and polymyxins [6].

Antimicrobial peptides (AMPs) have gained widespread attention as innovative drug candidates for treating these MDR infections. Typically comprising 10–50 amino acids with a positive net charge, AMPs exert bactericidal effects primarily through membrane targeting and disruption [7,8]. Unlike conventional antibiotics, cationic AMPs interact electrostatically with the negatively charged bacterial cell wall, leading to pore formation, membrane depolarization, and eventual cell lysis. While AMPs offer a broad spectrum of activity, their clinical translation has been limited by proteolytic degradation and rapid systemic clearance [9,10,11].

In our previous study, we developed a protease-resistant antimicrobial peptide, D-TN6, which demonstrated potent in vitro activity against reference strains and enhanced wound healing in vivo. Our prior characterization revealed that D-TN6 exerts rapid, dose-dependent bacterial membrane depolarization and structural disruption without inducing significant cytotoxicity or hemolytic activity. Furthermore, topical application of D-TN6 accelerated wound closure and reduced the bacterial load in acute infection models [12].

Building upon these promising findings, this study evaluated the clinical potential of D-TN6 against a panel of highly challenging MDR clinical isolates. Here, we report the potent antimicrobial efficacy of D-TN6 against 164 clinically relevant bacterial isolates, with a particular focus on strains resistant to last-resort agents like carbapenems and polymyxins. Additionally, we demonstrated the peptide’s high barrier to resistance development through serial passage assays, highlighting its potential as a therapeutic candidate in addressing the growing crisis of antimicrobial resistance.

## 2. Results

### 2.1. D-TN6 Activity Against ATCC Strains

**Figure 1:**
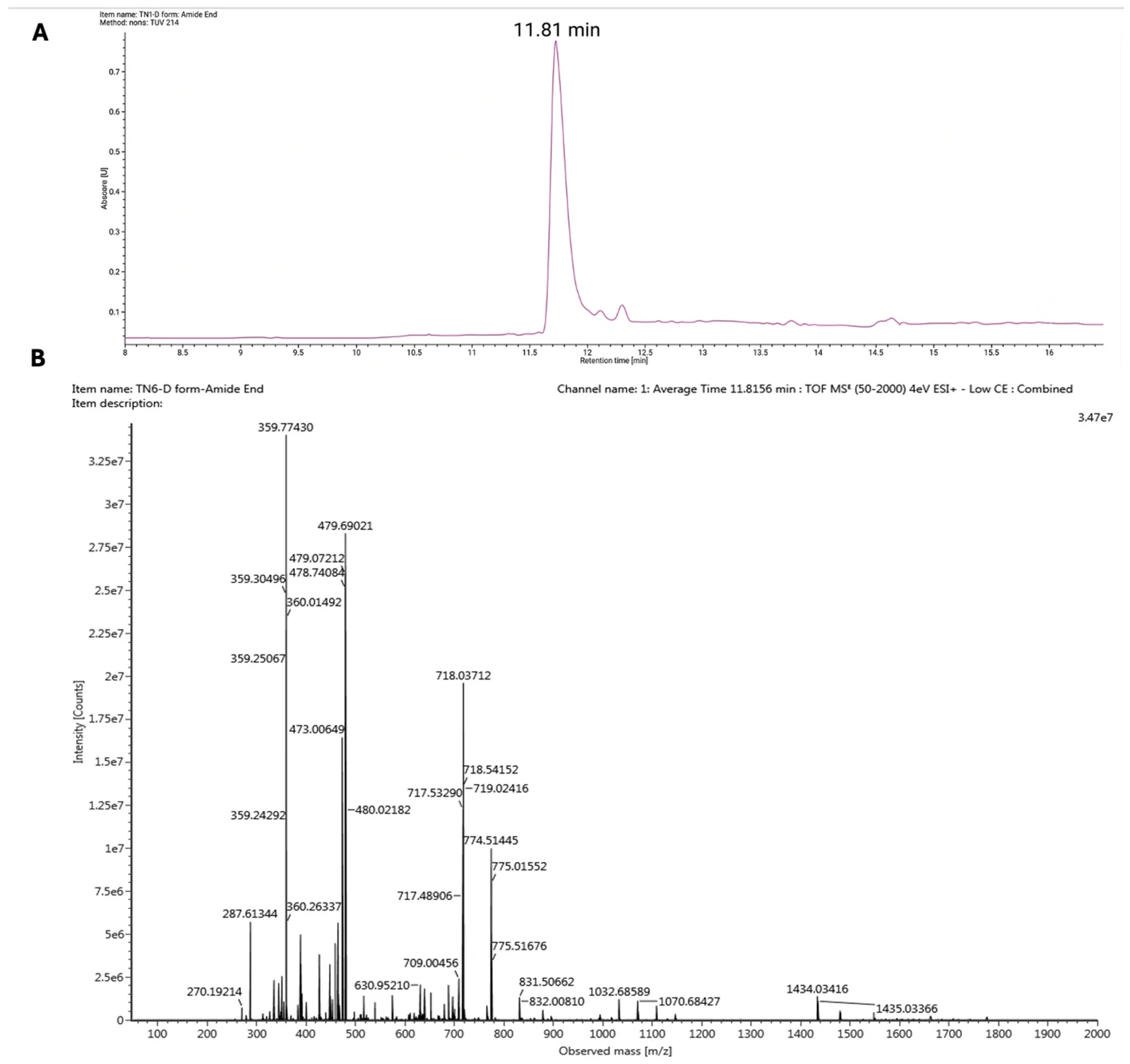
Characterization of the D-TN6 peptide: (**A**) UPLC chromatogram and (**B**) mass spectrum of D-TN6.

The amino acid sequence of the D-TN6 peptide, previously designed by Kocagoz et al. [12], is provided in Table 1. The peptide was synthesized and characterized by UPLC, confirming a purity > 90% at a retention time of 11.81 min (Figure 1A). The mass spectrum of D-TN6 confirmed its identity (Figure 1B).

### 2.2. Minimum Inhibitory Concentrations (MICs) of D-TN6 Against MDR Bacterial Isolates

The efficacy of the antimicrobial peptide D-TN6 was previously established using antibiotic-susceptible ATCC strains, encompassing both bacterial and fungal targets. Building upon these preliminary findings, the present study evaluated the efficacy of D-TN6 against a total of 164 clinical isolates of *E. coli* and key ESKAPE pathogens (Table 2). The majority of these isolates exhibited an MDR phenotype, the details of which are summarized in the tables for each pathogen.

All tested MDR *E. faecium* isolates were resistant to vancomycin and included strains with additional resistance to ampicillin, gentamicin, and linezolid, including a pan-resistant phenotype. Despite these complex resistance profiles, D-TN6 maintained consistent and potent activity across the entire group. The peptide demonstrated narrow MIC ranges with uniform MIC_50_ and MIC_90_ values, indicating robust performance against these high-priority clinical targets regardless of their baseline antibiotic resistance (Table 3).

A total of 17 *S. aureus* isolates were identified as MRSA and benzylpenicillin-resistant isolates. Additionally, various isolates displayed resistance to daptomycin, tetracycline, levofloxacin, and fosfomycin, indicating a MDR phenotype. D-TN6 demonstrated substantial therapeutic potential, maintaining high potency even among MDR strains (Table 4). Notably, the peptide exhibited remarkably low and uniform inhibitory concentrations across all MRSA isolates, indicating that its efficacy is not affected by existing resistance mechanisms to conventional antibiotics.

Among the isolates tested, 53 out of 60 *K. pneumoniae* strains were confirmed as extended-spectrum beta-lactamase (ESBL) producers and exhibited resistance to ceftazidime, ciprofloxacin, ertapenem, polymyxin B, and meropenem. Despite this extensive resistance profile, the antimicrobial peptide D-TN6 maintained stable inhibitory activity (Table 5). The consistent MIC_50_ and MIC_90_ values demonstrate the peptide’s capability to bypass established enzymatic and membrane-mediated resistance mechanisms.

Twelve of the tested *A. baumannii* isolates exhibited resistance to ceftazidime, ciprofloxacin, meropenem, and polymyxin B. Despite this high-level resistance, the antimicrobial peptide D-TN6 maintained activity against all isolates (Table 6). These results indicate that D-TN6 retains potent in vitro activity against *A. baumannii* strains that are resistant to multiple last-line therapeutic agents, including the membrane-targeting polymyxin B.

The tested MDR *P. aeruginosa* isolates displayed resistance to ceftazidime, ciprofloxacin, meropenem, and polymyxin B. These isolates were characterized by resistance to β-lactam antibiotics and/or the cell-membrane-disrupting polymyxin B. Despite these high-level resistance profiles, D-TN6 maintained stable and potent inhibitory activity across all tested isolates. Notably, the peptide bypassed established resistance mechanisms even in polymyxin B-resistant and pan-drug-resistant strains, demonstrating consistent efficacy regardless of the baseline antibiotic susceptibility (Table 7).

The tested *E. coli* isolates were predominantly composed of ESBL-producers with significant co-resistance to ceftazidime and ciprofloxacin. Despite the loss of efficacy in both β-lactam and fluoroquinolone classes among these isolates, D-TN6 maintained stable and potent inhibitory activity. The consistent MIC_50_ and MIC_90_ values demonstrate promising efficacy against ESBL-positive strains, respectively, confirming that D-TN6 retains potent activity against fluoroquinolone-resistant and ESBL-producing *E. coli* (Table 8).

### 2.3. Resistance Development Studies Against Antibiotics

**Figure 2:**
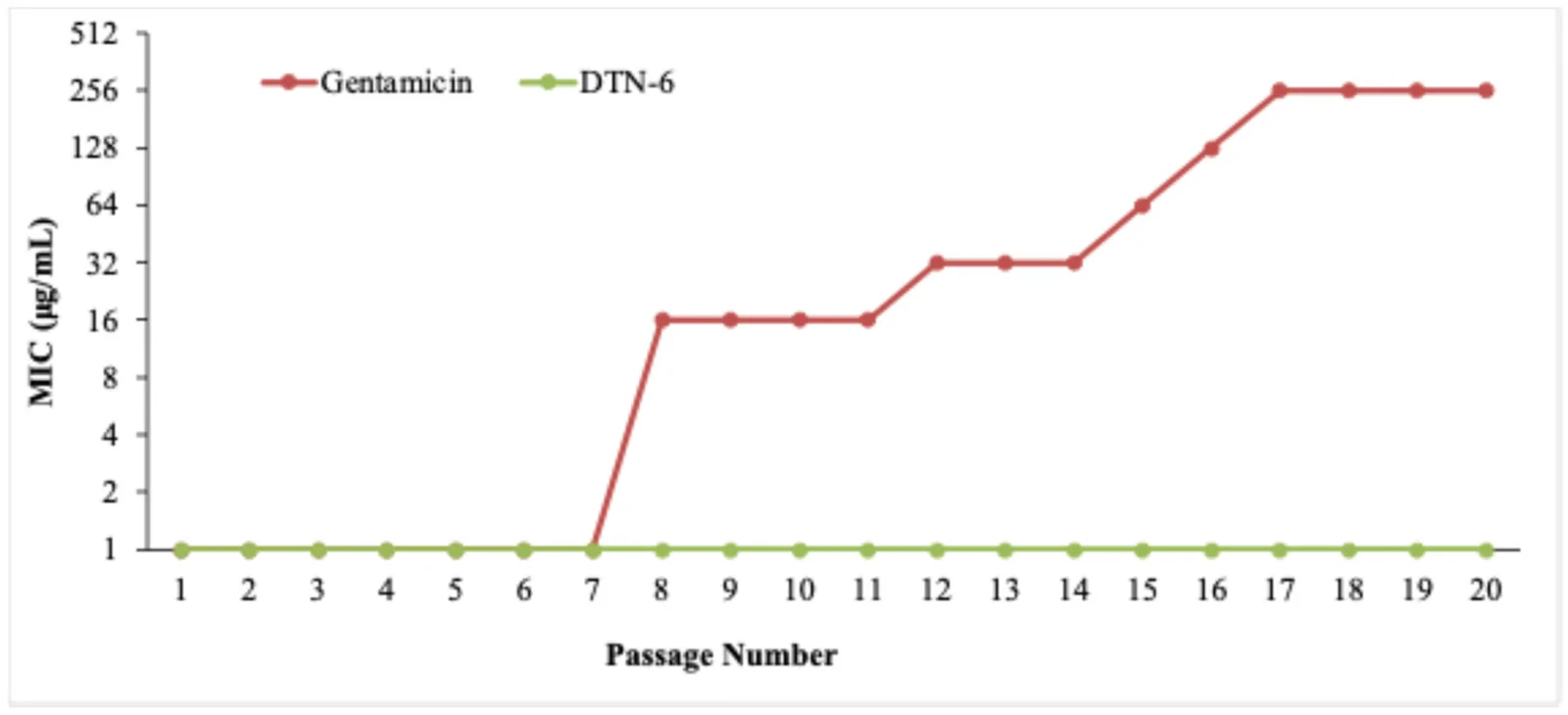
Variation in the MIC values of gentamicin (red line) and D-TN6 (green line) due to resistance development over 20 consecutive passages. Data points represent the mean values obtained from independent biological triplicates. Untreated growth controls were included in all assays to verify cell viability, and detailed passage conditions followed standard subculture protocols.

*E. coli* ATCC 25922 was employed to evaluate the potential for resistance development against gentamicin, an aminoglycoside antibiotic, and the D-TN6 peptide. For gentamicin, the initial MIC was 1 µg/mL. However, a marked increase in MIC was observed starting from the seventh passage, reaching 16 µg/mL. Subsequently, the MIC values rose exponentially, reaching 256 µg/mL by the 17th passage, at which point the resistance level stabilized (Figure 2). This significant upward trend confirms the rapid emergence of high-level bacterial resistance to gentamicin under selective pressure.

In contrast, D-TN6 exhibited a consistent MIC value of 1 µg/mL across all 20 consecutive passages, with no detectable increase observed throughout the experimental period. This sustained susceptibility indicates that the bacteria did not develop detectable resistance to D-TN6 under these conditions (Figure 2).

## 3. Discussion

In our previous study, we found that D-TN6 exhibited potent antimicrobial activity against the ATCC reference strains, with MIC values ranging between 0.5 and 2 µg/mL (Table 1). The cytotoxicity of the peptide was assessed using murine 3T3 fibroblasts and human HaCaT keratinocytes. Based on the HaCaT-derived selectivity indices (SI = IC_50_/MIC), D-TN6 demonstrated the lowest SI against *P. aeruginosa* (9.82), followed by *E. coli* and *S. aureus* (19.64 each). In contrast, the highest SI was observed against *Candida albicans* (39.28). Even at concentrations up to 16 μg/mL, the hemolytic activity of D-TN6 on human erythrocytes resulted in less than 50% lysis (HC_50_), which is at least eight times higher than its MIC against *P. aeruginosa* and 16 times higher than its MIC against *S. aureus* and *E. coli.*

Potentiometric measurements using TPP^+^-selective electrodes demonstrated that D-TN6 effectively permeabilizes the outer membrane and induces rapid, dose-dependent depolarization of the bacterial plasma membrane. The structural degradation of the membrane was confirmed by electron microscopy. Scanning electron microscopy (Thermo Scientific-Quattro SEM, Waltham, MA, USA) analysis revealed surface-level damage, such as pitting and blistering, while transmission electron microscopy (Thermo Scientific-Talos L120C, TEM, Waltham, MA, USA) imaging showed severe internal disruption, including cell membrane thickening and the formation of balloon-like filamentous protrusions from the cytosol, which ultimately led to cell lysis.

In the rat full-thickness excision wound model, topical application of the D-TN6 (at 40-fold MIC) significantly enhanced the rate of wound closure on days 4 and 6 compared to the saline-treated control group. Histopathological analysis revealed a more regular organization of the epidermal and dermal layers in D-TN6-treated rats, while biochemical assessments showed that the peptide prevented glutathione (GSH) depletion in the wound tissue. In a surgical site infection model involving sutures contaminated with *S. aureus*, topical treatment with D-TN6 resulted in a statistically significant reduction in the viable bacterial load within the wound tissue within 3 h post-application. These findings demonstrate that D-TN6 exerts a potent and rapid bactericidal effect in vivo, even under acute infection conditions that simulate clinical surgical scenarios [12].

Our previous work clearly demonstrated the potent antimicrobial activity of the D-TN6 peptide, including its established in vivo efficacy on a rat skin model. To evaluate the scope of this efficacy against high-priority clinical targets, the peptide was evaluated against MDR and pan-resistant *E. coli* and key ESKAPE pathogens, which are a group of clinical isolates that are particularly challenging to treat in hospital settings. These results highlight D-TN6’s capacity to target high-priority antibiotic resistance, demonstrating potent inhibitory effects where conventional treatments are often ineffective.

The principal finding of this work is the consistent antimicrobial profile of D-TN6 against 164 clinical isolates, including highly resistant *E. coli* and key ESKAPE strains. While many of these isolates were non-susceptible to last-resort agents such as vancomycin and polymyxins, D-TN6 demonstrated stable, potent activity throughout the panel. This underscores the D-TN6’s potential to serve as a sustainable alternative in treating life-threatening infections caused by pan-drug-resistant bacteria.

*E. faecium* is a Gram-positive pathogen implicated in a wide range of infections. Treatment has been increasingly challenged by the rise of VRE and MDR strains resistant to ampicillin, gentamicin, and linezolid. This MDR phenotype significantly limits therapeutic options. Ampicillin, a β-lactam antibiotic targeting bacterial cell wall synthesis, has long been the primary treatment for infections caused by *E. faecium* [13]; however, resistance compromises standard therapy and is frequently associated with hospital-adapted, high-risk lineages. Additionally, resistance to gentamicin and linezolid eliminates both synergistic aminoglycoside-based combinations and last-resort protein synthesis inhibitors [14]. Notably, a triple-resistant strain was inhibited by D-TN6 at 4 μg/mL.

*S. aureus* is a major Gram-positive pathogen responsible for a broad spectrum of infections ranging from skin and soft tissue infections to life-threatening systemic diseases. The increasing prevalence of MRSA has led to its classification in the high-priority group of the WHO Bacterial Priority Pathogen List [5]. In this study, MRSA isolates exhibited resistance to benzylpenicillin, a β-lactam antibiotic that inhibits bacterial cell wall synthesis by targeting penicillin-binding proteins. While several isolates exhibited a diverse resistance profile with different underlying mechanisms, D-TN6 demonstrated potent in vitro activity against these MDR strains. The development of resistance to daptomycin, a lipopeptide that disrupts the bacterial cell membrane via calcium-dependent insertion, threatens one of the few remaining bactericidal agents [15]. Unlike daptomycin, AMPs like D-TN6 disrupt membrane integrity by forming pores, a physical mechanism that makes the development of resistance significantly more difficult [12]. This is supported by the low MIC_50_ and MIC_90_ values obtained against daptomycin-resistant bacteria.

*K. pneumoniae* is a major Gram-negative pathogen frequently associated with nosocomial pneumonia, bloodstream infections, and urinary tract infections. The rapid emergence and spread of ESBL-producing *K. pneumoniae* is a critical public health concern, as these enzymes hydrolyze cephalosporins [16]. In addition to β-lactam resistance, ESBL-producing *K. pneumoniae* strains frequently exhibit co-resistance to other antimicrobial classes such as fluoroquinolones, aminoglycosides, and sulfonamides, further limiting therapeutic options [17,18]. In the present study, *K. pneumoniae* isolates exhibited extensive resistance profiles. Despite these extensive profiles, D-TN6 demonstrated notable activity. The efficacy of D-TN6 against strains resistant to carbapenems and polymyxins is particularly significant. A key advantage of D-TN6 is its membrane-targeting mechanism which provides several advantages over conventional antibiotics that target specific enzymes or biosynthetic pathways—such as reduced likelihood of resistance development and effectiveness against metabolically dormant cells [12]. Of note is its activity against polymyxin B-resistant isolates, the ability of D-TN6 to inhibit these strains indicates that it may bypass established barriers, although its long-term stability requires further investigation. These findings highlight the potential of D-TN6 as a promising therapeutic candidate for treating severe infections caused by ESBL-producing, pan-drug-resistant *K. pneumoniae*, and support further investigation into its clinical applicability.

*A. baumannii* is a Gram-negative pathogen that has emerged as a major cause of hospital-acquired infections. The rapid acquisition of resistance to β-lactams, fluoroquinolones, carbapenems, and even last-line agents such as polymyxins has led to the emergence of pan-drug-resistant strains, which pose a serious therapeutic challenge [19]. Classified in the critical priority category of the 2024 WHO Bacterial Priority Pathogen List, carbapenem-resistant strains are associated with alarmingly high mortality rates, particularly in intensive care settings [5]. In our study, all *A. baumannii* isolates were resistant to at least one of the following antibiotics: ceftazidime, ciprofloxacin, meropenem, or polymyxin B, and seven of these strains were pan-drug-resistant. Despite this resistance profile, D-TN6 demonstrated strong in vitro efficacy. This supports the role of D-TN6 as a valuable therapeutic agent for severe nosocomial infections where last-line agents have failed.

*P. aeruginosa* is a Gram-negative pathogen that causes severe infections, particularly in immunocompromised patients. Carbapenem-resistant *P. aeruginosa* is classified in the high-priority group of the WHO Bacterial Priority Pathogens List [5]. Despite these challenging resistance profiles, the tested *P. aeruginosa* isolates remained highly susceptible to D-TN6. The peptide’s membrane-targeting mechanism is advantageous, as it bypasses common resistance pathways such as β-lactamase production, efflux pump overexpression, and target site modification.

The *Enterobacteriaceae* family comprises a wide range of Gram-negative bacteria, including *E. coli*. Cephalosporin-resistant *E. coli* is currently categorized in the critical-priority group of the WHO Bacterial Priority Pathogens List [5]. ESBL-producing *E. coli* have become a dominant cause of MDR Gram-negative infections worldwide, not only in hospitals but increasingly in community-acquired infections. Forty-four of these ESBL-producing *E. coli* strains were resistant to ceftazidime, which is a β-lactam antibiotic that inhibits cell wall synthesis. This combination of resistance limits the use of the standard regimen [20]. As shown in Table 8, D-TN6 demonstrated potent in vitro activity against all ESBL-positive and ceftazidime-resistant *E. coli* isolates. The peptide’s activity is particularly relevant because it is unaffected by β-lactamase-mediated resistance and bypasses DNA gyrase/topoisomerase mutations that confer fluoroquinolone resistance [21].

The development of resistance to gentamicin is consistent with previous reports indicating that prolonged or repeated exposure to conventional antibiotics often activates adaptive resistance mechanisms [22]. In contrast, the stable MIC profile of D-TN6 across successive determinations suggests that its mode of action may overcome common resistance pathways. This aligns with the findings of Kocagoz et al., who reported that the D-TN6 peptide targets the bacterial membrane, thereby reducing the likelihood of resistance development [12]. Collectively, these results highlight the potential of D-TN6 as a promising novel therapeutic agent. To provide more evidence, future studies should extend this evaluation to other bacterial species encompassing both Gram-positive and Gram-negative organisms, including key ESKAPE pathogens. Nevertheless, these laboratory-derived data do not guarantee that resistance will not emerge during the long-term or widespread clinical use of D-TN6.

## 4. Materials and Methods

### 4.1. Peptide Synthesis and Characterization

Peptide synthesis, purification, and sequence verification via peptide mapping were performed according to the methods described by Kocagoz et al. [12]. Briefly, the D-TN6 peptide was synthesized via solid-phase peptide synthesis using an automated peptide synthesizer (Liberty Blue, CEM, Matthews, NC, USA). The peptide sequence was verified using a UPLC system (Acquity H-Class Bio) coupled to a Xevo^®^ G2-XS QToF mass spectrometer (Waters, Milford, MA, USA) with an electrospray ionization (ESI) source, operated in the positive ion mode. To ensure experimental consistency, a single master solution of D-TN6 was prepared and utilized across all assays in this study. All analytical characterization data correspond to this validated stock.

### 4.2. Antimicrobial Susceptibility Testing and Resistance Phenotype Classification

Antimicrobial susceptibility testing of all clinical isolates was initially performed using the VITEK^®^ 2 system (bioMérieux, Marcy-l’Étoile, France), and results were interpreted according to the European Committee on Antimicrobial Susceptibility Testing (EUCAST) clinical breakpoints (v.16.1). Where recommended by EUCAST, resistance phenotypes were confirmed using reference phenotypic methods. Linezolid resistance in *Enterococcus faecium*, cefoxitin resistance for MRSA confirmation in *Staphylococcus aureus*, ESBL production in *Klebsiella pneumonia* and *Escherichia coli*, as well as carbapenem resistance in *Klebsiella pneumoniae* were confirmed by disk diffusion-based methods. Polymyxin B susceptibility of *Klebsiella pneumoniae*, *Acinetobacter baumannii*, and *Pseudomonas aeruginosa* was determined by broth microdilution, in accordance with EUCAST recommendations. MDR and pan-drug-resistant phenotypes were classified according to the standardized international definitions proposed by Magiorakos et al. [23].

### 4.3. Minimal Inhibition Concentration

Ethical approval for the use of human-derived resistant bacterial strains in this study was granted by the Acibadem Mehmet Ali Aydinlar University Medical Research Evaluation Board (ATADEK) (No: ATADEK-2025-01/17, 9 January 2025).

MDR bacterial strains, including vancomycin-resistant *Enterococcus faecium*, methicillin-resistant *S. aureus*, *Klebsiella pneumoniae*, *Acinetobacter baumannii*, *Escherichia coli*, and *Pseudomonas aeruginosa* were kindly provided by Acibadem Labmed Clinical Laboratories. Bacterial strains were subcultured on Mueller–Hinton agar (MHA, Oxoid), while Mueller–Hinton broth (MHB, Oxoid) was employed for the preparation of the bacterial inoculum, followed by overnight incubation at 37 °C.

The initial concentration of the D-TN6 in dH_2_O was adjusted to 1024 µg/mL using the Pierce Quantitative Fluorometric Peptide Assay, and subsequent serial dilutions were performed in MHB. The bacterial suspensions were standardized to 1 × 10^7^ CFU/mL in MHB. The bacterial inoculum and AMP solutions were combined in triplicate at a ratio of 5:95 µL (*v*/*v*) and incubated at 37 °C overnight [12,24,25].

For each bacterial species, MIC_50_ was defined as the minimum concentration of the antimicrobial peptide required to inhibit the growth of 50% of the tested isolates, whereas MIC_90_ represented the concentration required to inhibit 90% of the isolates. These values were derived from cumulative MIC distributions and were employed to assess the in vitro efficacy of the tested compounds against resistant bacterial populations.

### 4.4. Resistance Development Studies Against Antibiotics

*E. coli* ATCC 25922 strains were utilized to induce antibiotic resistance against gentamicin and D-TN6 peptide under identical experimental conditions. The starting concentrations of both agents were adjusted to a maximum of 1024 µg/mL and serially diluted to 0.5 µg/mL.

Subsequent MIC assays were conducted under the same conditions, using the bacterial subpopulation harvested from the well containing the highest concentration of the respective agent that permitted visible growth in the preceding passage. This process was iteratively repeated for 20 consecutive passages to assess the rate and extent of resistance development [26].

## 5. Conclusions

In conclusion, this study demonstrates that the D-TN6 peptide exhibits potent, broad-spectrum antimicrobial activity against a panel of 164 MDR clinical *E. coli* and key ESKAPE isolates, including strains resistant to last-resort antibiotics, such as carbapenems and polymyxins. The ability of D-TN6 to maintain low MIC values for 20 consecutive passages to assess the rate and extent of resistance development highlights its distinct advantage over conventional agents such as gentamicin. These findings suggest that the membrane-disrupting mechanism of D-TN6 effectively circumvents existing bacterial defense strategies and minimizes the emergence of resistances. Consequently, D-TN6 emerges as a promising therapeutic candidate for addressing the global challenge posed by MDR infections; however, further studies are required to evaluate its systemic efficacy and safety in vivo.

## 6. Patent

A patent application (No: 2025/001242) has been filed for the D-TN6 peptide in the name of Acibadem University.

## Figures and Tables

**Table 1:** Amino acid sequence of D-TN6 and its minimum inhibitory concentration (MIC) values (μg/mL) against ATCC reference strains.

Peptide	Sequence	*S. aureus* ATCC 29213	*E. coli* ATCC 25922	*P. aerginosa* ATCC 27853	*C. albicans* ATCC 10231
D-TN6	RLLRLLLRLLR	1	1	2	0.5

**Table 2:** List of MDR bacterial pathogens and MIC values of D-TN6 peptide determined in this study.

Species	Total Number of sp.	D-TN6 MIC_50_ (μg/mL)	D-TN6 MIC_90_ (μg/mL)
*Enterococcus faecium*	13	4	4
*Staphylococcus aureus*	17	0.5	1
*Klebsiella pneumoniae*	60	1	8
*Acinetobacter baumannii*	12	4	4
*Pseudomonas aeruginosa*	11	1	1
*Escherichia coli*	51	1	4

**Table 3:** MIC values of D-TN6 against vancomycin-resistant *E. faecium* isolates.

Antibiotic	*n*	D-TN6 MIC Range (μg/mL)	D-TN6 MIC_50_ (μg/mL)	D-TN6 MIC_90_ (μg/mL)
Ampicillin	11	2–4	4	4
Gentamicin	7	2–4	4	4
Linezolid	1	4	NA	NA
Pan-resistant	1	4	NA	NA

*n*: total number of antibiotic-resistant species. NA: not applicable.

**Table 4:** MIC values of D-TN6 against methicillin-resistant *S. aureus* isolates.

Antibiotic	*n*	D-TN6 MIC Range (μg/mL)	D-TN6 MIC_50_ (μg/mL)	D-TN6 MIC_90_ (μg/mL)
Benzylpenicillin	17	0.125–2	0.5	1
Erythromycin	14	0.125–2	0.5	1
Daptomycin	13	0.125–2	1	2
Tetracycline	9	0.125–2	1	1
Levofloxacin	6	0.125–1	0.125	0.25
Fosfomycin	4	0.125–0.25	0.125	0.25
Mupirocin	2	0.125–0.25	NA	NA
Pan-resistant	3	0.125–2	NA	NA

*n*: total number of antibiotic-resistant species. NA: not applicable.

**Table 5:** MIC values of D-TN6 against MDR *Klebsiella pneumoniae* isolates.

Antibiotic	*n*	D-TN6 MIC Range (μg/mL)	D-TN6 MIC_50_ (μg/mL)	D-TN6 MIC_90_ (μg/mL)
ESB	53	0.25–8	1	8
Ceftazidime	52	0.25–8	1	8
Ciprofloxacin	43	0.25–8	1	8
Ertapenem	26	0.5–8	1	4
Polymyxin B	20	0.25–8	1	8
Meropenem	19	0.25–8	1	4
Pan-resistant	16	0.5–8	1	8

*n*: total number of antibiotic-resistant species.

**Table 6:** MIC values of D-TN6 against MDR *A. baumannii* isolates.

Antibiotic	*n*	D-TN6 MIC Range (μg/mL)	D-TN6 MIC_50_ (μg/mL)	D-TN6 MIC_90_ (μg/mL)
Ciprofloxacin	10	2–8	4	8
Ceftazidime	8	4–8	4	8
Meropenem	7	2–8	4	8
Polymyxin B	2	0.25–2	NA	NA
Pan-resistant	7	2–8	4	8

*n*: total number of antibiotic-resistant species. NA: not applicable.

**Table 7:** MIC values of D-TN6 against MDR *P. aeruginosa* isolates.

Antibiotic	*n*	D-TN6 MIC Range (μg/mL)	D-TN6 MIC_50_ (μg/mL)	D-TN6 MIC_90_ (μg/mL)
Ciprofloxacin	9	0.5–2	1	1
Meropenem	7	0.5–2	1	2
Polymyxin B	6	1–4	1	4
Ceftazidime	5	1–2	1	2
Pan-resistant	2	1–2	NA	NA

*n*: total number of antibiotic-resistant species. NA: not applicable.

**Table 8:** MIC values of D-TN6 against MDR *E. coli* isolates.

Antibiotic	*n*	D-TN6 MIC Range (μg/mL)	D-TN6 MIC50 (μg/mL)	D-TN6 MIC90 (μg/mL)
ESBL positive	49	0.25–8	1	4
Ceftazidime	44	0.25–8	1	1
Ciprofloxacin	31	0.25–4	1	4
Pan-resistant	27	0.25–4	1	4

*n*: total number of antibiotic-resistant species.

## Data Availability

The original contributions presented in this study are included in the article. Further inquiries can be directed at the corresponding authors.

## Abbreviations

The following abbreviations are used in this manuscript:

MIC	Minimum inhibition concentration
MDR	Multidrug-resistant
AMP	Antimicrobial peptide
MRSA	Methicillin-resistant *S.aureus*
VRE	Vancomycin-resistant *E. faecium*
